# Evaluation of Permanent Growth Hormone Deficiency (GHD) in Young Adults with Childhood Onset GHD: A multicenter study

**DOI:** 10.4008/jcrpe.v1i1.7

**Published:** 2008-08-05

**Authors:** Merih Berberoğlu, Zeynep Şıklar, Feyza Darendeliler, Şükran Poyrazoğlu, Şükran Darcan, Pınar İşgüven, Aysun Bideci, Gönül Öcal, Rüveyde Bundak, Bilgin Yüksel, İlknur Arslanoğlu

**Affiliations:** 1 Ankara University, Faculty of Medicine, Pediatric Endocrinology Unit, Ankara, Turkey; 2 Istanbul University, Faculty of Medicine, Pediatric Endocrinology Unit, Istanbul, Turkey; 3 Ege University, Faculty of Medicine, Pediatric Endocrinology Unit, Izmir, Turkey; 4 Gazi University, Faculty of Medicine, Pediatric Endocrinology Unit, Ankara, Turkey; 5 Cukurova University, Faculty of Medicine, Pediatric Endocrinology Unit, Adana, Turkey; 6 Düzce University, Faculty of Medicine, Pediatric Endocrinology Unit, Düzce, Turkey; 7 Goztepe Research and Education Hospital, Istanbul, Turkey; +90−312 595 67 91+90−312 319 14 40zeynepsklr@gmail.comAnkara Üniversitesi Tıp Fakültesi Pediatrik Endokrinoloji Bilim Dalı, Cebeci− Ankara/Turkey

**Keywords:** Growth hormone deficiency, childhood, retesting

## Abstract

**Background**: Reconfirming the diagnosis of childhood onset growth hormone deficiency (GHD) in young adults is necessary to demonstrate the need for continuation of GH therapy.

**Objective**: This nationally−based study was planned to establish GH status during adulthood in childhood−onset GH deficient patients and to evaluate factors that would predict persistency of the GHD.

**Methods**: In this multicenter study, 70 GH deficient patients who had reached final height were evaluated after completion of GH treatment. Fifty−two patients (74%) had isolated GHD and 18 patients (26%) had multiple pituitary hormone deficiency (MPHD). Patients who had reached final height and the pubertal Tanner stage 5 were reevaluated for GH status. After at least 6 weeks of cessation of GH treatment, patients were retested with insulin induced hypoglycemia.

**Results**: GHD was found to be transient in 64.3% of all patients. Of the isolated GH deficient patients 82.7% had transient GHD, whereas 88.9% of the MPHD patients showed persistent GHD. Comparison of isolated GH deficient and multiple hormone deficient patients indicated higher peak GH, IGF−I and IGFBP−3 levels in isolated GH deficient patients. No parameter was significantly different in the transiently and persistently GH deficient patients with respect to gender. Although specificity of IGF−I value of less than −2 SD showing persistency of GHD was lower than the specificity of IGFBP−3 value of less than −2 SD (65.7% vs 84%), negative predictive values were similar for the two parameters (85.2% and 84%, respectively).

**Conclusion**: Most of the cases of childhood onset GHD are idiopathic and the GHD is transient. In patients with MPHD, GHD is generally permanent. Low IGF−I and IGFBP−3 levels are supporting findings to show persistency of the GHD.

**Conflict of interest:**None declared.

## INTRODUCTION

It has been shown in several studies that a normally functioning hypothalamus−pituitary− insulin like growth factor I (IGF−I) axis is very important not only in children but also in adults.

Adult growth hormone deficiency (GHD) is characterized by decreased lean body mass (LBM) and bone mineral density (BMD), increased visceral adiposity, abnormal lipid profile and diminished quality of life.(1, 2) Therefore, some patients with childhoodonset GHD might need to continue growth hormone (GH) replacement therapy after attainment of final height. However, most of the childhood−onset GHD patients, when retested, do not have classical severe GHD as adults. The high incidence of normal GH responses on retesting has been shown in patients with idiopathic or isolated GHD.(3) However in patients with multiple pituitary hormone deficiency (MPHD) and/or organic etiologies, persistence of severe GHD into adulthood is more frequent (>90%).(4) Reconfirmation of the GHD diagnosis will be necessary in order to demonstrate the need for continuation of GH therapy.

In this multicenter study we evaluated GH stimulation tests and serum IGF−I and IGF binding protein (IGFBP)−3 levels in a group of patients who had been diagnosed with GHD in childhood and had reached final height after GH treatment. The study aimed to evaluate the frequency of GHD in adults with previous childhood−onset GHD, to identify the potential patients for GH treatment during transition and adulthood. and also to evaluate factors that would predict persistent GHD.

## MATERIALS AND METHODS

**Figure 1:**
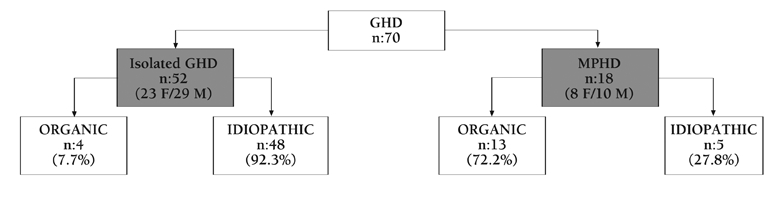
Distribution of the patients by etiology of GHD

This study was conducted in five centers in Turkey. Patients who had been diagnosed as having idiopathic or organic isolated GHD or MPHD before the completion of their growth were included in the study. GHD diagnosis was based on a peak GH level <10 ng/mL in two pharmacological tests. GH treatment was discontinued in these patients when growth velocity during the previous year decreased to less than 2 cm and the bone age had reached >14 years in girls, >16 year in boys, and after completion of puberty. GH secretion was reevaluated in a total of 70 (31 F/39 M) subjects who had completed the pediatric GH treatment protocol. Their main clinical findings and etiologies are summarized in Table 1 and Figure 1. Data regarding final height will not be discussed in the present paper. Weight and height were measured by standard methods and body mass index (BMI) calculated accordingly.

In all patients GH secretion was reevaluated by insulin tolerance test (ITT) at least 6 weeks after discontinuation of the replacement treatment. Serum IGF−I and IGFBP−3 concentrations were determined at the same time. Insulin (0.05 U/kg) was given intravenously at time zero and venous blood samples for GH determination and glucose were obtained at time 0 and at 30, 60, 90 and 120 minutes after the injection. If GH peak during the test was less than 3 ng/mL, the patient was diagnosed to have severe permanent GHD.

Serum GH (ng/mL) levels were measured by Immunoradiometric assay (IRMA) using Immunotech^®^ kit. Serum IGF−I (ng/mL) levels were measured by Immunoradiometric assay (IRMA) using Immunotech^®^ kit and serum IGFBP−3 (ng/mL) levels were measured by Immunoradiometric assay (IRMA) using DSL^®^ kit.

Statistical analysis was done by using Minitab^®^ software package program. P<0.05 was considered statistically significant. Comparisons between groups were performed using Student is t−test. Spearman r coefficient was used to correlate IGF−I, IGFBP−3, and peak GH after retesting. Specificity, sensitivity, positive predictive value, and negative predictive value of IGF−I levels of less than −2 SD and IGFBP−3 value of less than −2 SD were determined by standard statistical methods.

Standard deviation scores (SDS) were calculated for IGF−I and IGFBP−3 by reference to a normal population in order to control for age and gender.(5)

Informed consent was obtained from the patients.

**Table 1:**
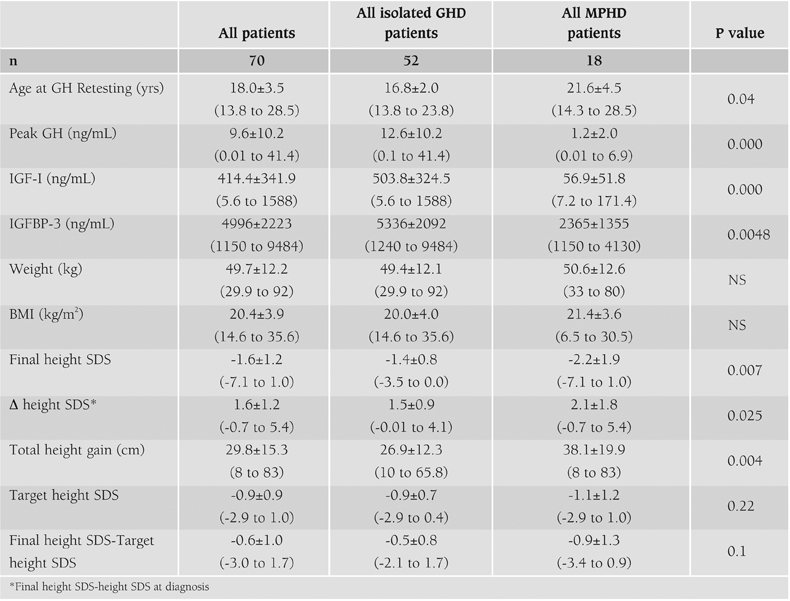
Clinical characteristics of the subjects included in the study

## RESULTS

As seen in Figure 2, 74% of GH deficient patients had isolated GHD. By taking a peak GH value of less than 3 ng/mL in the ITT test, 9 patients (17.3%) were found to have persistent GHD and 43 (82.7%) to be transiently GH deficient. On the other hand, among patients with MPHD only 2 patients (11.1%) were transiently GH deficient.

Overall 64.3% of patients were transiently, and 35.7% of patients were persistently GH deficient. Characteristics of the groups with isolated GHD or MPHD are shown in Table 1. Patients who had isolated GHD achieved their final height at younger ages. In concordance with a lower peak GH response, IGF−I and IGFBP−3 levels were significantly lower in the multiple hormone deficient groups (p<0.05) and inversely, height improvement was better (Table 1).

The groups of children with persistent or transient GHD were subdivided into males and females. None of the parameters differed significantly with respect to gender. There were significant positive correlations between peak GH and IGF−I, and IGFBP−3 levels in all patients (Figure 3) (IGF−1 r=0.297, p=0.036; IGFBP−3 r=0.45, p=0.03).

The IGF−I and IGFBP−3 SDS values were lower in the group that had peak GH values lower than 3 ng/mL (Table 2).

When the cut−off was taken as −2 SD, specificity and sensitivity of IGF−I in confirming persistency of GHD were 65.7% and 73.3% respectively. Its positive predictive value and negative predictive value were 33.3% and 85.2%, respectively. For IGFBP−3, specificity and sensitivity were 84%, and 60% respectively. The positive value and negative predictive values were 60%, and 84%, in the same order.

Finally, while the negative predictive values were high for both of these parameters, an IGFBP−3 value below −2 SD was found to be more specific than an IGF−I value below −2 SD.

**Figure 2:**
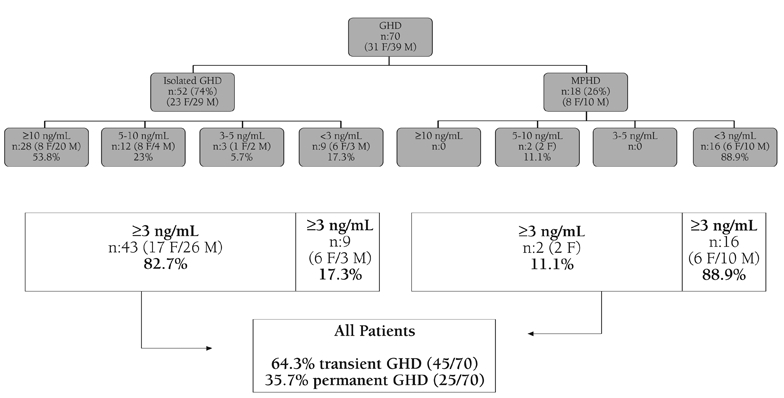
GH responses after ITT retest in isolated or multiple GH deficient patients

**Figure 3:**
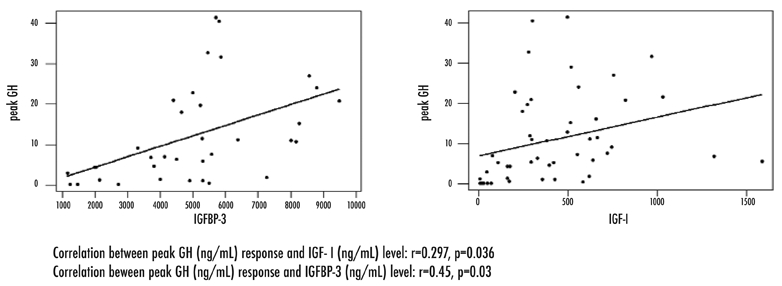
Correlation between peak GH response and IGF−I and IGFBP−3 levels

**Table 1:**
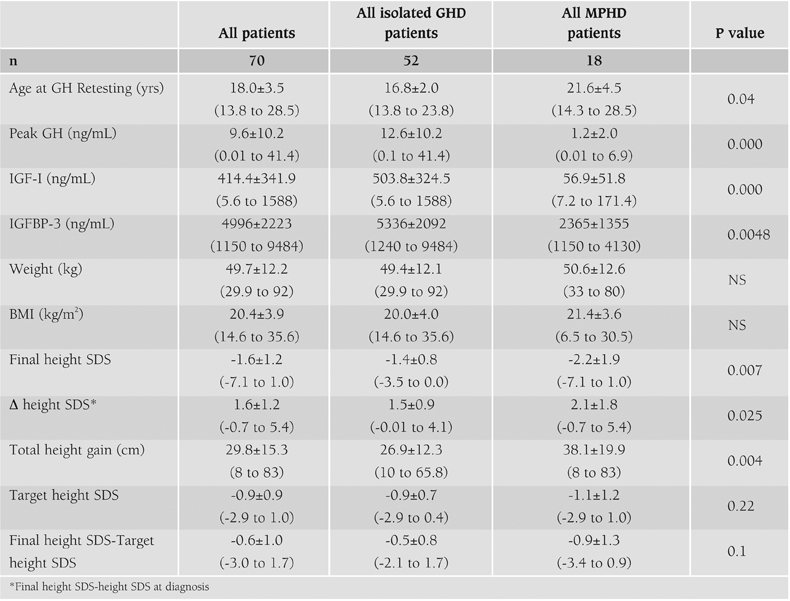
Clinical characteristics of the subjects included in the study

**Table 2:**
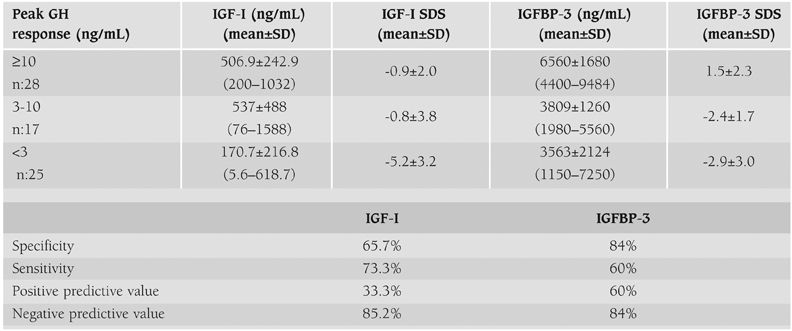
IGF−I and IGFBP−3 levels according to peak GH response

## DISCUSSION

Data from recent studies suggest that in patients diagnosed to have organic GHD in childhood, when GH is discontinued at final height in adolescence, a cascade of symptoms and signs attributable to the deficiency of the metabolic effects of GH may be experienced in adulthood.(6) A great number of studies have shown that the incidence of idiopathic and isolated GHD is much higher in childhood. The majority of children with GHD when retested as adults do not have the classical severe GHD.(7) This high incidence (70%) of normal GH responses on retesting has been shown in patients with idiopathic and isolated GHD.(3) In compliance with the published reports, this study has also shown a higher proportion of transient GHD (82.7%) in patients with idiopathic isolated GHD (Figure 2). By contrast, the incidence of transient GHD was only 11.1% in organic MPHD. This finding indicates that the organic etiologies are often severe and can be assumed to be permanent at the beginning of the therapy.

In this study comparison of patients with isolated GHD and with MPHD indicated a better Δ height SDS and total height gain in the multiple pituitary hormone deficient patients. No parameter was statistically significant when compared on the basis of gender in patients with transient and persistent GHD. Our data confirm that there are no auxological and clinical signs to predict the transiency or the persistence of GHD except for a history of organic disease and presence of MPHD. Toogood et al(8) have also reported that the severity of hormone deficiency in adults is related to the degree of hypopituitarism.

Meanwhile the question of how to confirm the diagnosis of adult GHD in an adolescent patient who has completed linear growth is still being debated.

It is well known that during childhood and adolescence, GH secretion is higher than in adulthood. Individual laboratory values of GH deficient patients can overlap with those of normal controls. The Growth Hormone Research Society guidelines suggest a peak GH response on ITT of less than 3 ng/mL as being diagnostic of GHD in adulthood.(9) It is not clear whether this value can confirm adulthood GHD exactly. Usually patients with MPHD have peak GH levels less than 3 ng/mL. Hence in our study we used this cutoff value to confirm permanent deficiency. On the other hand the patients who had peak GH cut−off values between 3−5 ng/mL might be GH deficient as well. In fact, in the transition period in late adolescence a cut−off value of 5 ng/mL is advocated for the diagnosis of persistent GHD and continuation GH therapy because adolescents have higher GH levels than adults.(7) In our study the 3 additional patients with peak GH level between 3−5 ng/mL in the isolated GHD group in late adolescence can be considered as candidates for GH therapy in the transition period. There were no similar cases in the MPHD group. The prognosis of patients with a GH response of 5−10 ng/mL is not known. However, deterioration of GH secretion can occur in this group therefore it may be advisable to follow these patients.(10)

In adults ITT appears to be the gold standard test of choice.(9, 11) Because of the risks associated with hypoglycemia, currently GHRH−arginine test is an alternative to ITT.(12) Because of the difficulties in finding GHRH in our country, we performed ITT on all patients in experienced centers.

In young adults in whom retesting is necessary, a period off GH is required before testing. Consensus statements and clinical practice guidelines suggest 1−3 months between GH discontinuation and retesting.(13) In this study we retested at least 6 weeks after GH discontinuation. The optimal duration of washout before retesting serum GH and IGF−I, IGFBP−3 levels are not clear. Longer period may be risky in the follow−up of those adolescent patients.

Zucchini et al showed that one third of GHD subjects diagnosed before puberty presented with normal GH secretion at puberty. According to this study, puberty seems the most likely time for normalization of GH secretion. In all patients we performed retesting after completion of puberty.

IGF−I levels are not useful as the sole diagnostic test for many GH deficient patients. Levels vary between and within individuals and may not be helpful for making a definitive diagnosis of persistent GHD. However, in contrast to patients with adult onset GHD, very few patients with childhood− onset GHD have IGF−1 or IGFBP−3 levels within normal ranges.(15) In compliance with the published reports, there were significant positive correlations between peak GH and IGF−I, and IGFBP−3 levels in all our childhood onset GH deficient patients. Lower GH response and lower IGFI, and IGFBP−3 levels in multiple pituitary hormone deficient groups appear to be characteristic of any degree of childhood onset hypopituitarism. Furthermore lower IGF−I and IGFBP−3 SDS values are associated with the persistent GHD group who had peak GH response below 3 ng/mL. In our study the specificity of IGF−I for persistent GHD was lower than of IGFBP−3 (65.7% vs. 84%), However, negative predictive values were higher and similar for these two parameters (85.1% and 84% respectively). This finding means that normal IGF−I and IGFBP−3 levels highly exclude the diagnosis of GHD. Baum et al showed that no patient with GHD had IGF−I levels in the upper half of the normal range. On the other hand, based on specificity and sensitivity values, the use of serum IGF−I and IGFBP−3 alone to predict GHD cannot be recommended. Nevertheless,those patients with organic MPHD could be excluded from retesting.

In conclusion, most patients with childhood onset GHD are idiopathic and GHD is frequently transient in this group of patients. Generally GHD is persistent in patients with MPHD. We strongly offer retesting of all patients with idiopathic and isolated GHD patients to identify transient patients after they have reached their final height. Low IGF−I and IGFBP−3 levels are supportive findings to show persistency and severity of GH deficiency. It appears that only longitudinal followup can demonstrate those patients who may or may not need GH treatment in adult life.

**Figure 2:**
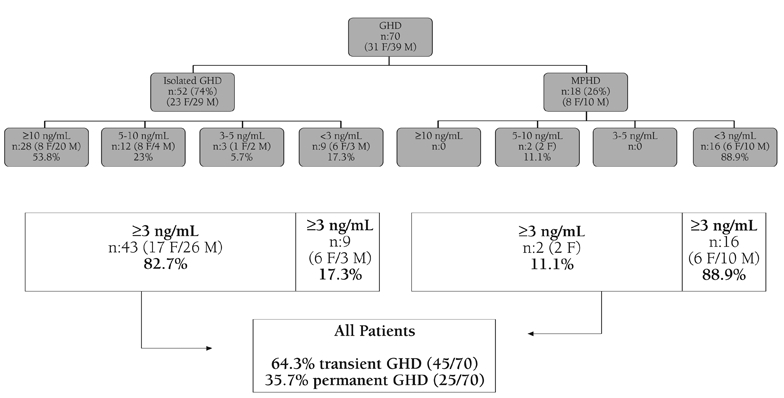
GH responses after ITT retest in isolated or multiple GH deficient patients

## ACKNOWLEDGEMENT

The authors thank the following doctors for contributing data: Ismail Yildiz, Damla Gökflen, Ediz Yesilkaya, Peyami Cinaz
